# Experiences with Casting Metals

**Published:** 1911-01-15

**Authors:** C. N. Thompson

**Affiliations:** Chicago


### EXPERIENCES WITH CASTING METALS.

BY C. N. THOMPSON, D.D.S., CHICAGO

The real vital part of the casting process is vested in the wax impression, sprue, casting ring and base. Whether the gold is best forced into place by air or steam pressure, vacuum or centrifugal force is yet an open question and I am not prepared to discuss it though a combination of air pressure and vacuum in one machine would seem to be most direct.

But the details connected with handling these appliances including investment materials, in order to reproduce wax models accurately, using alloys of gold that give the least shrinkage, sufficient malleability to burnish easily, yet enough rigidity to withstand all necessary stress and in cases of bridge piers good stiffness, taking into consideration the casting of plates, crowns, bridge intermediates and fillings, will require time to decide. But it is certain already that the matter of using the proper alloys as required by varying conditions is of great importance and that gold of unknown karat cannot be used indiscriminately.

### IMPRESSION WAX.

The wax impression has been discussed so freely in the journals that we could almost pass it, yet the ideas about heating and handling it are so varied that we must stop long enough to say that none but the surface of the wax should be really soft. The main body should be about as hard as the fingers will handle, with the outer surface softened quickly immediately before using and if possible be confined with some sort of matrix leaving, in case of fillings, at least a film of wax remaining over the margins. Most wax is too soft for accurate work, requiring too much cold to harden it. Yet some are so brittle and chill so quickly afterwards that perfect adaptation is almost out of the question and carving without reheating is next to impossible. A good guide is to choose one that will get quite hard in the mouth without chilling if given time, and for all work out of the mouth something not so hard is best as it is easier handled and can be chilled to suit. Brittleness in impression wax is always a menace, it should be hard but tough. In case of fillings there are some good features about taking an impression of cavities and working from a model, mainly though at the present time because in case of failure in casting, another can be made. Yet it looks as if when the right material for a model can be secured we shall all come to it for inlays.

### THE SPRUE.

The general dimensions of the ideal sprue must depend somewhat upon the machine in which it is to be used. Air pressure machines require that the casting ring shall be of nearly uniform length which necessitates sprues of various lengths in order that wax models of various sizes may be placed central in the ring but a sprue of uniform length may be used in the others by using a casting ring of the proper length to suit the case. This seems best as it gives uniform distance from the crucible bottom to the impression in all cases.

The sprues furnished with some machines seem coarse and fit only for heavy pieces, for gold in its transfer from the crucible to mold needs a passage somewhat in proportion to the size of the case, therefore an assortment of sprues to correspond somewhat with the various sized pieces of work would be desirable and increase exactness. The larger and shorter the sprue the less heat and pressure will be required to cast sharp and because of the inefficiency of our investment materials the less of both the better will be the result.

Long, slender sprues remove the mold too far, giving the metal time to chill in transit; the nearer the mold can be to the crucible without endangering caving in the top of the investment the less force will be required to transfer the gold from one to the other. Yet the escape of air from the mold is equally necessary, and is best facilitated by having the mold as near the bottom as is safe against bulging, for as the air must escape through the porosity of the investment the shorter the distance the easier will it release. So casting rings of varying lengths are necessary in order that the mold outlines may be equally distant from both top and bottom, though the same result may be easily gained by the use of an instrument resembling the casting ring base to press into the bottom of the ring while investing, hollowing it somewhat like the crucible on the opposite end thus holding the impression central.

The end of the sprue upon which the wax is fastened in order to give the greatest security with the least heat or pressure in attaching should be made like a tenon to fit a mortise, that is, made perfectly flat across the end yet having a thin feather of metal about 1-32 of an inch long protruding from it. This when heated in a very small flame in order to heat the very tip only, imbeds into the wax to the shoulder easily and cools quickly lessening distortion and prevents the wax from turning during investment.

It is found good practice whenever possible and practical to imbed the sprue in the impression before removing it as it makes a convenient attachment and tends toward accuracy because of one less handling.

A sprue with a fine hole drilled central from end to end that will admit of a small brass wire that can be removed and attached to the impression facilitates handling by attaching it while the wax is in place either in the cavity or elsewhere and returning the wire into its place in the sprue before investing. This makes a dainty way of handling small pieces.

### CASTING RING.

For investment materials of the present day there is a call for an inside formation of some kind to prevent the investment from slipping out of the ring in handling, due mostly to the ring expansion under heat. This arrangement usually takes the form of a groove, chime rim at the bottom, or a gradual taper of the ring. The groove seems most practical and if this guard could be arranged near the end of the ring opposite to the crucible it would answer every purpose, be easier to keep clean and be below the stress where it would prevent cracking. The frequency with which cracked investments occur when this offset is placed on a level with the wax model suggests that the pressure incident to the casting process was sufficient to force the investment below the groove downward and produce a crack at that point, for if a ring will expand in circumference sufficient to loosen its contents, it will also expand lengthwise and protrude beyond the investment slightly which being held central gives room for the investment below to drop slightly under pressure and occasionally cause a crack.

### CASTING RING BASE.

The general form of this part is determined by the requirements of each of the various machines or methods and is usually what it must be to fit conditions and as long as it gives a crucible form to hold the molten gold and a free-throated exit into the sprue track giving a good seat for the sprue and ring and being well polished there is little more to ask. However, the proper depth of the crucible seems mostly over-done tending to unnecessarily pocket the blowpipe flame and in the case of some to overheat the pressure pad above it, and as a suggestion, a crucible only large enough to hold the melted slug a little more than half its depth would seem fair. At any rate I find it an improvement to broaden the top of the investment crucible by trimming off the corner. This, however, is with the understanding that the casting ring stands above investment as for air or steam pressure. The sprue seat in the base could easily be made adjustable by having a screw follower and in this way lengthen or shorten the sprue at will, but it is hard to keep clear and destroys the finish in the investment at the union of the crucible and sprue track, which is important.

### INVESTMENT MATERIALS.

The adage, "that fools will sometimes enter where angels fear to tread," seems applicable to the subject at this time, for who knows? we have tried most of them, found few of them worthless, none of them perfect; yet as they are the best we have what are the results?

The writer once stopped to listen to an investment manufacturer, and a good man too, who was extolling one of his make for inlay purposes, the chief virtue of which was that it did not change form under heat and when asked if it did not expand at all he, in turning to answer, saw Dr. Prothero and his answer was "I do not know."

The truth is that during a series of experimental tests, the writer found it very difficult to get anything like uniform results and it will probably be some time before we will get what we want, so the best that I can do is to pass along to the finished casting and work back.

After cleaning the cast piece the first thought is that it is very rough and a magnifier reveals two things: first, that there are always little air spaces in the investment; second, that there are numerous little pits and prominences and that usually most of the pits are on what was the bottom of the cast as it stood in the machine. This latter means that gravity had something to do with it and that our investment materials are too loosely constructed, for a great part of the imperfections to be found in finished work are caused by particles from the inner walls of the mold being displaced by the movements produced by heat expansion. These particles collect upon the bottom of the mold as it stands in the machine, causing pits by displacing the gold and prominences in the places they vacated. Such disturbances are probably caused at the time of most intense heat action, which is the casting period and the jarring incident to the action of applying the machine. Vacuum machines here hold the palm because of the smoothness of their action.

Careless or rapid heating and over-heating are also causes of these defects, all moisture and vapor should be given time to escape without bubbling or vigor of any kind, as the movements of boiling wax and accumulated water in the mold must loosen particles that afterward settle where they are not wanted and should never occur. If during the warming up period for ordinary work the investments are inverted, that is with the crucible down, part at least of the wax and moisture will escape early and harmlessly. But for fine work the investment should be set upon asbestos paper and have the top of the ring covered giving plenty of time at a heat below the boiling point until dry. This procedure allows the hot asbestos to absorb the wax and moisture from the bottom and the cover prevents action upward that might disturb the interior of the mold.

Over-heating destroys the bond in the investment, allowing particles to become detached and rattle out of place. Cracks also finally form from over heating. A well set dry and clean mold is necessary, the hotter the better provided that it is very quickly heated and below a semblance of red. I find myself using less heat on the investment with experience.

Excessively hot gold in casting may easily destroy an otherwise good mold by burning the exposed protruding points on the inside as seen by the rocking of fillings upon prominences in cavities. This in the opinion of the writer marks the passing of the oxy-hydrogen blowpipe as used today, as it is so very easy in its use to unnecessarily overheat the metal and a gas blowpipe can easily be timed to give about what is necessary.

The writer's experience at this time is that the best and smoothest casts are secured by first painting the wax impression, sprue, and the casting ring base with a very thin layer of an exceedingly fine powdered non-fusing investment material, afterward completing the investment with a coarse hard open material. This gives smoothness to the piece where it is needed without clogging the air spaces throughout the entire investment.

### AIR IN THE MOLD.

The resistive action of the air in the mold when casting by pressure is a factor not given due consideration, and as the question of vent is dependent upon the porosity of the investment it looks as if a tight investment would forestall a clear cast and it is my experience that it will.

Air pressure machines with a solid metal bottom ring and using the usual investment compound will not cast sharp, whereas with the same conditions and an open bottom ring with a vent cut in base will cast with little pressure This being true, the investment must always contain vent holes between the particles of the investment for the escape of air which will always be sufficient to create an irregular surface to all castings, so the finer the particles of the investment material next to the mold the better, and the coarser balance of the investment gives free vent. The most desirable finish to a casting is that it may be perfectly smooth, sharp and clear, and as retentive roughness is necessary it can easily be created on the surfaces desired by the use of a saw, bur or stone. A roughened exterior to a cast piece means pitted margins as well as on other parts.

Smoothness is dependent upon several things: one of the most noticeable is that the alloys that oxidize seem not only to cast with a poor surface but adhere to the particles of investment as though fused and are hard to clean, and the lower the karat the more marked is this tendency; besides, the alloys seem to volatilize and if hot enough give off gas and seldom cast perfectly.

Alloys of bench scraps if worked out with the oxy-hydrogen blowpipe repeatedly and pickled hot between will finally become pure enough to use in general bench work but not for inlays. When burning alloys out of gold of unknown karat by the use of the oxy-hydrogen blowpipe there is ever danger of explosion if the gold is poured into sulphuric acid. It should cool until it can be lifted with pliers and boiled if necessary. Borax has no place in the crucible as it will cast with the gold, producing pits in the cast.

Pure gold or gold alloyed with platinum or iridium, being free from oxid, usually casts clear and for fillings gives good results. At the present time for fine work such as inlays an alloy of 24 parts of pure gold and one part of platinum previously alloyed with 20 per cent of iridium is giving good results for inlays and seems to be of good resistance without being hard and is what is used for all inlays or bridge piers, though inlays for the latter if large and unsupported by a post are always made as a cavity lining only, filling them to the desired bulk later with clasp metal and solder.

Intermediates for bridge work are more accurate if cast separately, then assembled and soldered. Bridges of two or more piers should be made in sections and soldered together afterwards. Porcelain teeth for which pieces are to be cast should be backed with either pure gold or platinum and the facings be either riveted or cemented after the final soldering. Long pin vulcanite cuspids can be used in making bridge intermediates by putting a square of gutta-percha over the pins while getting the impression and carving, afterward cementing them to place. Backs for long pin facings are cast using graphite pins to retain the pin holes during casting. Casting direct against porcelain teeth is admittedly impractical and it probably always will be because the difference in temperature of molten gold and an invested tooth is so great that fractures are sure to occur as a result of the shock and afterward from the shrinkage. The possibilities of casting against the facings is so alluring that all sorts of attempts have been made to succeed with it, but experience demonstrates that it is too risky to be practical; but as a result of these attempts we have many novel and pretty ways of casting for facings or loose-pin crowns for bridge intermediates and attachments for plates that obviate the necessity of taking this chance, making very durable and beautiful pieces.

Celluloid wherever possible can be used as matrix or bands with the knowledge that it will incinerate and leave a print that will cast clear. Its use is indicated in bands for roots wherein it is easily fitted and removed, saves time and obviates occasional soldering such as occurs when metal bands are used, and in the instance of the loose-pin crown with cast base the presence of the celluloid band supports the wax during the removal of the porcelain and pin, casting the base only, which is an advantage in fitting cast to the root and allows the use of a composition pin which is considered more serviceable than is one of platinum. Large proximal fillings if difficult can be neatly supported by using a celluloid band encircling the tooth and held snug with a tent. After the wax is in place, by the use of a hot instrument point it may be cut both labially and lingually, leaving that attached to the impression which will incinerate with the wax. This procedure facilitates making posterior bridge pier inlays of rigid metal.

Cast plates except as partials are not practical yet and even then there is a question that pertains more to the proper alloys to be used than whether it can be done or not.

The writer has not had sufficient experience in this class of work to make any definite suggestions as to the proper alloy for plates.

The idea of casting clasp metal with uniformity is found impractical because it partly loses its springy quality and so frequently assumes a crystallized condition that results are uncertain. When this crystallized condition occurs the addition of a small amount of pure gold may give a free casting rigid alloy again, but it is always uncertain and a fixed alloy of known karat is recommended. So far as I now know 22 karat gold alloys with platinum and iridium in the same proportions as pure gold and platinum for fillings proves best for partials, at any rate it casts clean and clear and seems to be about equal to clasp metal in stiffness, a little more iridium might be an advantage. Casting lower partial dentures using number 10 iridio platinum wire to connect from space to space and using loose-pin crowns for the teeth seems an ideal restoration. Cast the spaces separately and solder the connecting truss. Full dentures in cast gold would require a larger machine than we now have, but even then their practicability would be doubtful on account of waste; besides full dentures entirely of porcelain are coming.

### DISCUSSION.

Dr. W. T. Reeves: I have finally concluded that I get my best results using from three to four pounds of pressure. By holding my left hand on the cover I can close the mold and with my right turn the lever that controls the pressure, up to fifteen or twenty pounds. There is an interval of somewhere in the neighborhood of three seconds between the closing of the flask and the turning on of more pressure. In that way I feel that I get the gold into the mold without distorting it, and then I have the pressure back of that to hold the gold in place.

Dr. E. M. S. Fernandez: One of the minorities which has aided me in getting good castings, was to cut a few circular grooves and two cross-wise grooves on the metallic base which holds the flask while casting. This base, prepared in that way, allows a free exit of the air, thereby allowing the castings to fill the mold completely. Another important feature in this exit of air from the surrounding mold is to have two mixtures of investment. The first mixture of investment that surrounds the inlay should contain very fine silex, and the second mixture, which is to surround the first investment and fill the greater part of the flask, should contain coarser silex. The plaster, which is to some extent destroyed in the burning of the mold, will allow minute crevices which also permit the air to escape.

I beg to differ with the essayist in regard to the body of investment under the casting, which I believe he claims should be thin. If the second investment is of a coarser material, I prefer a pretty fair thickness of same.

In proximal cavities for inlay work, I believe it imperative to use matrices. The wax should not be too soft nor too warm when ready to be pressed into the cavity and the point of the wax which is to go into the cavity first should be slightly softer than the upper part, thereby allowing itself to be pressed firmly against the walls of the cavity and give good adaptations to the margins.

The size of sprue should correspond to the size of the casting—the smaller the casting, the smaller the sprue—and in very small castings I have had very satisfactory results by using an ordinary heavy pin as a sprue, but of course shorter in length. The thinner the sprue, the shorter in proportion.

Overheating of the mold must be avoided and a generous amount of gold should be used in order that the metal remain melted long enough to allow itself to be pressed into place before congealing.

I also agree with the essayist in regard to the ordinary gas blowpipe being preferable to the oxy-hydrogen blowpipe for gold casting.

In regard to the karat of gold to be used for the different classes of cast work, I beg to be permitted to say, that for inlays of 22 karat gold, and for crowns and bridge work, 22 karat gold with the addition of 1-6 22 karat gold solder has given me the most satisfactory results.

Dr. W. B. Tym: The taking of the impression may be simplified by starting with harder wax, and after having forced this as far as possible, if you have not the desired results, with a spatula, drop a small quantity of soft wax at the point where the impression is imperfect, the hard wax acting as a piston will force the softer wax into the desired position, thus giving and accurate impression.

The question as to size and length of the sprue should be governed entirely by the kind of machine used. The air, steam and centrifugal machines necessitate shorter sprues than do the vacuum machines. In the use of the vacuum machine, the sprue should be long enough to place the wax model to be reproduced in gold nearer the base of the casting ring, as the suction produced will relieve cavity of all air present, and if the metal is sufficiently heated it will be instantly drawn to place.

In the use of a large sprue the casting force must be applied the instant the flame is removed, because the gold has a tendency to fall into the sprue tract and may congeal sufficiently to cause the transfer from the crucible to the mold to become sluggish, resulting in a failure of definite outlines in the cast.

Dr. E. R. Carpenter: I originally took my impression in the same way Dr. Thompson speaks of, with a matrix, and it then became a personal equation with the operator. I find I can take my impression without a matrix in less time, and more accurately, and so naturally that is the course for me to pursue. I will give a short resume of my method. In the first place, in driving the wax home I hold it lingually-bucally a few minutes to keep it tight in the space, thereby forcing it lower down cervically, to get a good excess there; then the occlusion, of course, by biting and grinding on the wax. Then the excess is cut away lingually-bucally, and smoothed over with smooth spatulas or burnishers, whichever you prefer, and then I take a thin steel polishing strip and slide down through proximally, and cut out not all but part of the excess. Then you can take a very thin twilled silk tape, so thin that it will follow down the little space in which you put your polishing strip. That should be wide enough to cover the proximo-occlusal corner or angle, and in doing so comes up over. That should be lubricated, according to Dr. Taggart's last discovery, with *vegetable* oil. The inlay is drawn down and polished, then a sprue inserted near the proximo-occlusal angle, and chilled thoroughly. With a pair of light pliers grasping the sprue easily, the wax can be taken out of the cavity. I gain two or three advantages by doing it in this way. After the inlay is cast the sprue is cut off half way between the button and attachment of the inlay, and the spud is left there so that you can handle the inlay with your pliers. I think a great many accidents, especially in early work, have been caused from the lack of use of magnifying glasses, with which you see some of the minute peculiarities.

Dr. Thompson (closing the discussion): I am certain that there is as much damage done by gold above the fusing point as any other thing that we do. The proof of that is this: I have been in the habit of investing fillings in the center of the top, or on the contact point at one end, and when I invested on the occlusal surface I nearly always spoiled the fit by rocking of the piece in the center. When I invested at the contact point I had less of it, although it was not entirely eradicated. Then I thought, because some of the teeth were pulpless, that I would invest on the under side. I have never failed to get a fit in that direction. The pressure we have been using is far in excess of anything that is necessary, and I have been casting away down beolw five pounds, and the result has been good. The main advantage, after we have got the gold the proper heat, is to have the cavity heated as short a time, and as low a heat as possible, because every degree of heat causes a little more expansion, and shakes off pieces that create part of this roughness. If it is hollowed on the bottom and top it will heat very quickly under the blow pipe, and the main point I think, is to keep the air pressure down. Then you have no fear of bulging or crushing these investments that are so soft as soon as they are heated hot. Now the necessity for a smooth cast is apparent to all, and the smoothness of the cast depends on the fineness of the substance against which the gold goes, and it is my belief that if we could paint that with some substance that would be very fine, and as the wax disappears cement the parts together, and then cover it with something coarse and open, we would have very little resistance to the air, and could make the cast with low pressure. I figure that the nearer we can get the mold to the bottom the less air resistance there will be, and the nearer we can get it to the top the freer will be the transfer. I have felt that the pressure we have had is uncertain, and I wanted to get something that would go with a light touch, and go true, without straining, or hurting the parts.—December 1910, *Review*.

